# A Rare Manifestation of Hepatitis A Associated Cryoglobulinemia

**DOI:** 10.7759/cureus.36948

**Published:** 2023-03-31

**Authors:** Lakshmi Pappoppula, Syed Muhammad Hussain Zaidi, Peter A Iskander, Anthony Iskander, Beshir Saeed, Ayman Elawad, Mark M Aloysius, Vikas Khurana, Simin Nasr, Khalid Ahmed

**Affiliations:** 1 Internal Medicine, The Wright Center for Graduate Medical Education, Scranton, USA; 2 Internal Medicine, Xavier University School of Medicine, Oranjestad, ABW; 3 Internal Medicine, Jefferson Lansdale Hospital, Lansdale, USA; 4 Internal Medicine, Howard University Hospital, Washington DC, USA; 5 Gastroenterology, The Wright Center for Graduate Medical Education, Scranton, USA; 6 Family Medicine, The Wright Center for Graduate Medical Education, Scranton, USA

**Keywords:** immunoglobulins, vasculitis, steroids, cryoglobulins, hepatitis a

## Abstract

Cryoglobulins can precipitate in the blood when exposed to lower temperatures. These abnormal immunoglobulins are more commonly affiliated with Hepatitis C infection but there have been cases reported with Hepatitis A association for which we present this case. The patient was treated with steroids which did show gradual improvement of symptoms but, ultimately, the patient developed renal failure and required temporary hemodialysis. Care should be taken to assess patients with cryoglobulins for other viral serologies besides Hepatitis C.

## Introduction

Cryoglobulins are abnormal immunoglobulins (Igs) that precipitate at low temperatures and dissolve again upon warming [1]. Cryoglobulinemia refers to the presence of these cryoglobulins in the blood; this can lead to inflammation associated with purpura, neuropathy, arthralgias, and glomerulonephritis [2]. Hepatitis A virus (HAV) is a common infectious etiology of acute hepatitis worldwide. It is most commonly spread via the oral-fecal route such as through water, food, or close contact. Acute HAV infection is typically a self-limited illness characterized by nausea, vomiting, right upper quadrant abdominal discomfort, malaise, anorexia, myalgia, fatigue, and fever [3]. The prevalence of Hepatitis A infections has dropped by roughly 92% since the vaccine was introduced back in 1995; in the United States, however, it still makes up about 50% of the reported viral hepatitis cases. It was estimated that back in 2009, there were more than 21,000 cases of Hepatitis A in the United States, and greater than 1.4 million worldwide [4]. Hepatitis C has been described as the main diagnosis to consider in patients with mixed cryoglobulinemia. Other hepatotropic viruses, including Hepatitis A, have been noted, but these have been very rare [5]. Cutaneous vasculitis is another extrahepatic complication of Hepatitis A that has been described in the literature [6].

## Case presentation

A 58-year-old female with a past medical history of diabetes mellitus type II, hypothyroidism, and hypertension was admitted with complaints of bloating and abdominal pain with fullness for about a week. She reported recent travel to Arizona three months prior and denied any sick contacts. She was hemodynamically stable at the time of presentation. Physical examination was notable for scleral icterus and mildly distended, but non-tender, abdomen. The remainder of the physical examination was unremarkable. The initial lab workup is as follows (Table 1).

**Table 1:** Table depicting the progression of the patient’s lab values from admission to discharge.

	Latest Reference Range and Units	Day 0	Day 11	Day of Discharge
Sodium	135-146 mmol/L	133	134	131
Potassium	3.5-5.1 mmol/L	4.2	4.6	5.4
Chloride	98-107 mmol/L	94	94	90
Carbon Dioxide (CO_2_)	22-32 mmol/L	16	21	23
Blood Urea Nitrogen (BUN)	6-20 mg/dL	32	64	78
Creatinine	0.5-1.0 mg/dL	2.8	7.3	7.3
Estimated Glomerular Filtration Rate	>=60 mL/min	19	6	6
Anion Gap	7-15 mmol/L	23	19	181
Glucose	70-120 mg/dL	143	130	165
Calcium	8.4-10.2 mg/dL	8.5	7.7	9.3
Magnesium	1.5-2.6 mg/dL	2.3	2.8	2.5
Phosphorus	2.5-4.8 mg/dL	5.3	5.2	6.7
Protein	6.0-8.3 g/dL	6.2	6.0	6.2
White Blood Cell (WBC)	4.00-10.80 K/uL	9.38	6.07	15.9
Hemoglobin (Hgb)	12.0-15.3 g/dL	14.4	9.8	10.3
Hematocrit	36.0-45.2%	45.3	30.2	32.1
Mean Corpuscular Volume (MCV)	81.5-97.5 fL	84.4	83.2	86.5
Platelets	140-400 K/uL	168	141	223
Albumin	3.8-5.0 g/dL	3.3	2.9	2.8
Aspartate Amino-transferase (AST)	10-35 U/L	5,228	2,478	63
Alanine Transaminase (ALT)	10-35 U/L	4,792	3,398	76
Alkaline Phosphatase	35-130 U/L	313	276	189
Bilirubin, Total	<=1.2 mg/dL	7.9	8.8	20.5

**Figure 1:**
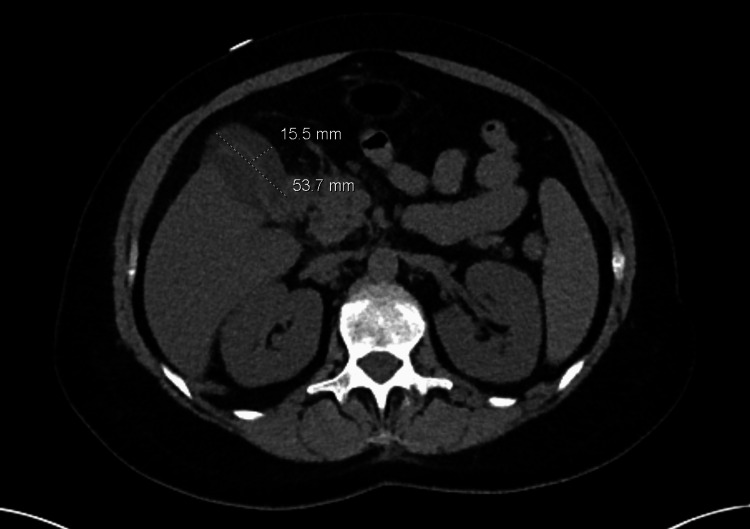
CT A/P showing a fluid collection adjacent to the gallbladder measuring up to 1.6 x 5.4 cm. CT: computed tomography; A/P: anterior-posterior

The urine toxicology screen was negative. Urinalysis showed amber-colored, cloudy-appearing urine that was positive for bilirubin, protein, blood, WBC, a few RBCs, and amorphous crystals. Right upper quadrant ultrasound showed a small hepatic lobe cyst and findings concerning cholecystitis with possible gallbladder perforation/peri-cholecystic phlegmon. This was followed up with a computed tomography (CT) of the abdomen/pelvis which showed hepatic steatosis, a left hepatic tiny cyst, and findings suggestive of perforated acute cholecystitis with either a focal peri-cholecystic fluid or a small collection adjacent to the gallbladder measuring up to 1.6 x 5.4 cm, colonic diverticulosis, reactive peri-portal lymph nodes and atherosclerotic changes in vessels (Figure 1).

The decision was made to admit the patient secondary to acute hepatitis and was started on intravenous (IV) fluids and IV Zosyn. She was evaluated by the Surgery team for suspicion of acute perforated cholecystitis picture noted on imaging. No acute surgical intervention was indicated, per their recommendations, as she had no gallstones; clinical signs/symptoms of cholecystitis, and was suspected to be due to primary liver disease. A gastroenterology consult was also made for which they noted a differential diagnosis to include viral infections, ischemic liver injury, drug-induced liver injury/toxin-induced, autoimmune hepatitis, common bile duct (CBD) obstruction, and Budd Chiari. Doppler ultrasound was done which ruled out portal vein thrombosis. Magnetic resonance imaging (MRI) liver showed an abnormal appearance of the gallbladder including marked wall thickening/edema with small peri-cholecystic fluid. No biliary dilatation/choledocholithiasis was noted. This was further evaluated by a hepatobiliary iminodiacetic acid (HIDA) scan which showed liver dysfunction and severe cholestasis with no biliary transit visualized.

She underwent further infectious work including acute viral hepatitis panel (Hepatitis A/B/C), serology for herpes simplex virus, cytomegalovirus, and autoimmune hepatitis workup including anti-smooth muscle antibody, anti-mitochondrial antibody, rheumatoid factor, SS/Ro, SS/La, antinuclear antibody, and total immunoglobulin levels. This came back positive for Hepatitis A IgM antibody suggesting acute Hepatitis A infection. Complement levels for C3 and C4 were low at 22 mg/dL (normal: 90-180 mg/dL) and 4 mg/dL (normal: 10-40 mg/dL) respectively. The cryoglobulin screen was positive while the cryocrit was less than 5% (within normal limits). She was then managed conservatively off of antibiotics. During the course of hospitalization, she also required insulin drip for better glycemic control likely due to sepsis causing uncontrolled hyperglycemia. Labs were monitored daily which showed gradual improvement in her aspartate aminotransferase (AST)/alanine transaminase (ALT) and up-trending total bilirubin. Nephrology was consulted for the worsening of her kidney function who ultimately started on hemodialysis.

Rheumatology also evaluated the patient who started on IV Methylprednisolone 1 gm daily for three days followed by oral Prednisone 30 mg twice daily. She continued to remain on insulin drip support for better glycemic control. Furthermore, she underwent a renal biopsy which came back negative for immune complex-mediated renal disease. Over the course of hospitalization, her liver enzymes gradually normalized while bilirubin and creatinine remained elevated. She was set up with outpatient hemodialysis and discharged home with follow-up appointments with Nephrology, Rheumatology, and Endocrinology. Three months post-discharge, dialysis was discontinued and her repeat blood work was as follows (Table 2).

**Table 2:** Table depicting follow-up labs after discharge.

	Reference Range	Follow-up Labs Three Months after Discharge
Bilirubin, Total	<=1.2 mg/dL	1.4
Aspartate Amino-transferase (AST)	10-35 U/L	23
Alanine Transaminase (ALT)	10-35 U/L	17
Alkaline Phosphatase	35-130 U/L	120
Blood Urea Nitrogen (BUN)	6-20 mg/dL	12
Creatinine	0.5-1.0 mg/dL	0.8
Estimated Glomerular Filtration Rate	>=60 mL/min	89

## Discussion

Cryoglobulinemia is a condition where abnormal immunoglobulins precipitate in serum at temperatures below 37°C; they dissolve upon rewarming. Cryoglobulins can deposit in the blood various vessels causing obstruction/vasculitis and can therefore involve various organs. These vasculitides are immune-complex-mediated. Vasculitis is mostly prevalent in mixed cryoglobulinemia (types II and III) which is usually associated with Hepatitis C; rare cases have been noted, however, with Hepatitis A [1]. Signs and symptoms of Hepatitis A infection can include nausea, vomiting, fever, and abdominal pain (to include a few). Interestingly enough, it has been associated with an aversion to cigarettes and alcohol. The severity of symptoms is generally proportional to age [4]. Other atypical symptoms that have been noted can include but are not limited to, fulminant liver failure, autoimmune hepatitis, or cholestasis [7]. Extra-hepatic and unusual complications reported include acalculous cholecystitis, hemolysis, acute renal failure, reactive arthritis, new-onset diabetes, preterm labor, and pericardial and pleural effusions [8]. Other manifestations have been described that include evanescent skin rash and transient arthralgias. Although rare, there have been some documented cases of arthritis and cutaneous vasculitis have been associated with cryoglobulinemia in literature [9]. Two studies involving relapsing Hepatitis A complicated by arthritis and cutaneous vasculitis showed evidence of cryoglobulinemia in both, with cryocrit values of 4.3% and 8.6% respectively. Serologic studies showed that these cryoglobulins consisted of polyclonal IgM and IgG [10]. The diagnosis of acute Hepatitis A is clinical. It can be confirmed with positive HAV IgM antibodies and the presence of jaundice or cholestasis greater than three months [7]. Our patient was diagnosed with Hepatitis A with significantly elevated transaminase and a viral panel. Autoantibody workup including rheumatoid factor, ANA, SS/Ro, SS/La, anti-smooth muscle antibody, and anti-mitochondrial antibody was sent despite no improvement in clinical symptoms which was negative. Although the Rheumatoid factor was negative it does appear to enhance the deposition and pathogenicity of immune complexes [10]. Cryoglobulinemia has been reported as being present during the acute phase of Hepatitis A and resolves as the liver disease regresses [11]. Immune response to Hepatitis A can last up to 25 weeks with higher anti-HAV titers in serum correlating to longer courses; at times it can be difficult to show the viral antigen from target organs [10]. Different intrinsic mechanisms of the virus have been proposed. One theory is that the host's immune system is unable to produce the necessary antibodies to neutralize the virus in which case the cryoglobulins are the result of interactions between the virus and the host [12]. Our patient had renal failure requiring hemodialysis for which cryoglobulin deposition may have contributed despite the biopsy being negative for cryoglobulin vasculitis. In HCV-negative patients, cryoglobulinemia with pulmonary and gastrointestinal involvement, renal insufficiency, and age >65 years are independently associated with increased mortality [13]. The patient did require oral steroids for treatment which were effective in reducing the atypical manifestations of acute infection, but it is yet to be applied in the adult population [12].

## Conclusions

Rare extrahepatic manifestations of Hepatitis A have been described in the literature. Cryoglobulinemia presents via continuous active stimulation to produce immunoglobulins and can result in vasculitis and other various cryoglobulinemia-related symptoms. In addition to Hepatitis C, it is prudent to check for the HAV in patients with liver disease who develop systemic vasculitis manifestations. It is unclear at this time if steroids play a beneficial role in the adult population or if the long-term complications are the same as described in Hepatitis C-related cryoglobulinemia. Regardless, in our patient, we have an established complication of renal failure. There have not been many reported cases showing a relation between Hepatitis A and cryoglobulinemia; further investigation should be made, therefore, on extrahepatic manifestations of Hepatitis A.
